# Changing Epidemiology of Respiratory Tract Infection during COVID-19 Pandemic

**DOI:** 10.3390/antibiotics11030315

**Published:** 2022-02-25

**Authors:** Hung-Jen Tang, Chih-Cheng Lai, Chien-Ming Chao

**Affiliations:** 1 Department of Medicine, Chi Mei Medical Center, Tainan 71004, Taiwan; 8409d1@gmail.com; 2 Department of Internal Medicine, Kaohsiung Veterans General Hospital, Tainan Branch, Tainan 710, Taiwan; dtmed141@gmail.com; 3 Department of Intensive Care Medicine, Chi Mei Medical Center, Liouying, Tainan 73657, Taiwan

**Keywords:** antibiotic resistance, COVID-19, non-pharmaceutical intervention, respiratory tract infection, SARS-CoV-2

## Abstract

The outbreak of COVID-19 has significantly changed the epidemiology of respiratory tract infection in several ways. The implementation of non-pharmaceutical interventions (NPIs) including universal masking, hand hygiene, and social distancing not only resulted in a decline in reported SARS-CoV-2 cases but also contributed to the decline in the non-COVID-19 respiratory tract infection-related hospital utilization. Moreover, it also led to the decreased incidence of previous commonly encountered respiratory pathogens, such as influenza and *Streptococcus pneumoniae.* Although antimicrobial agents are essential for treating patients with COVID-19 co-infection, the prescribing of antibiotics was significantly higher than the estimated prevalence of bacterial co-infection, which indicated the overuse of antibiotics or unnecessary antibiotic use during the COVID-19 pandemic. Furthermore, inappropriate antimicrobial exposure may drive the selection of drug-resistant microorganisms, and the disruption of infection control in COVID-19 setting measures may result in the spread of multidrug-resistant organisms (MDROs). In conclusion, NPIs could be effective in preventing respiratory tract infection and changing the microbiologic distribution of respiratory pathogens; however, we should continue with epidemiological surveillance to establish updated information, antimicrobial stewardship programs for appropriate use of antibiotic, and infection control prevention interventions to prevent the spread of MDROs during the COVID-19 pandemic.

## 1. Introduction

Although it has been more than 2 years since the first outbreak of severe acute respiratory syndrome coronavirus 2 (SARS-CoV-2) in Wuhan, China, the negative impact of coronavirus disease 2019 (COVID-19) on global health is continuing [1]. To date, more than 376 million people have been infected and 5 million have died in the whole world [2]. However, effective antiviral agents against SARS-CoV-2 remain limited [3]. In addition to pharmaceutical agents and vaccines against COVID-19, many stringent non-pharmaceutical interventions (NPIs), such as universal masking, hand hygiene, social distancing, the isolation of patients with COVID-19, contact tracing, lockdown, travel restrictions, and cancelation of mass gatherings, have been developed and implemented worldwide to prevent SARS-CoV-2 transmission and contain the COVID-19 outbreak [4,5]. Despite the implementation of the abovementioned NPIs not being able to completely stop the spread of COVID-19, these measures did result in a significant reduction in the prevalence of many other infectious diseases, particularly respiratory tract infections [6,7,8,9,10,11].

Before the emergence of COVID-19, pneumonia, including community-acquired pneumonia (CAP) and hospital-acquired pneumonia (HAP)/ventilation-associated pneumonia (VAP), was one of the most common infectious diseases, and could cause major health problems, associated with high morbidity and mortality in all age groups worldwide [12]. According to the estimation of the Global Burden of Diseases (GBD) study in 2016, 336.5 million cases of lower respiratory tract infections (LRTIs) developed. The estimated incidence was 32.2 per 100,000 people worldwide and these cases caused more than 2.3 million deaths [13]. In 2019, the incidence of LRTI showed increases—there were 489 million incident cases of LRTI, and 11 million prevalent cases of LRTI [14]. Many kinds of microorganisms, including bacterial, viral, and fungal, have been reported as pathogens of pneumonia; however, the causative microorganisms for CAP and HAP differ substantially. The most common causative pathogens in CAP are *Streptococcus pneumoniae*, respiratory viruses, *Haemophilus influenzae* and other atypical bacteria such as *Mycoplasma pneumoniae* and *Legionella pneumophila*. In contrast, the most frequent microorganisms in HAP are *Staphylococcus aureus* (including both methicillin-susceptible *S. aureus* (MSSA) and methicillin-resistant *S. aureus* (MRSA)), Enterobacterales, *Pseudomonas aeruginosa*, and *Acinetobacter* spp. [15,16,17]. However, most of this knowledge was based on studies prior to the COVID-19 pandemic and the emergence of SARS-CoV-2 could significantly change the whole picture of respiratory tract infections in many ways, including epidemiology, microbiological distribution, antibiotic consumption, and antimicrobial resistance. Therefore, we conducted this review to provide an update and comprehensive information about respiratory tract infections during the COVID-19 pandemic.

## 2. Epidemiology

The implementation of NPIs to control COVID-19 had indirectly and largely reduced hospital utilization due to CAP. In Japan, a study based on claims data from the Quality Indicator/Improvement Project database in 262 hospitals reported that the number of hospitalizations for CAP during the period between March and July 2020 drastically decreased by 48.1% compared with the same period in 2019 [18]. In addition, milder cases showed a greater decrease in the year-over-year ratio than severe ones: mild −55.2%, moderate −45.8%, severe −39.4%, and extremely severe −33.2% [18]. In Hong Kong, all-cause pneumonia hospitalization during the COVID-19 pandemic significantly decreased by 17.5% (95% CI 16.8% to 18.2%, *p* < 0.0005) [19]. In Taiwan, a retrospective national epidemiological surveillance study using the electronic database of the Taiwan National Infectious Disease Statistics System also demonstrated a significant decrease in cases of all-cause pneumonia during the COVID-19 pandemic [20]. For elderly patients, a population-based study in Italy using Tuscany healthcare system data showed that compared with the average of the corresponding periods in 2017–2019, significant reductions of 31.5% in weekly hospitalization rates for CAP were observed from the week in which the national containment measures were imposed (week 10) until the end of the first COVID-19 wave in July 2020 (week 27) [21]. However, the rates of ICU admission and in-hospital mortality were significantly higher among these elderly patients with CAP during the pandemic than the previous period [21]. Children were similarly affected due to COVID. In Brazil, one study based on the Department of Informatics of the Brazilian Public Health System database showed that there was a significant reduction of 82% in the average incidence of hospitalizations due to pneumonia for children ≤14 years (897.4/100,000 during pre-pandemic vs. 162.1/100.000 during pandemic, incidence rate ratio = 0.18; 95% CI, 0.15–0.21) [22]. In Poland, Grochowska et al. noted an 81% decline in LRTI-associated hospital admissions among pediatric patients using ICD-10 analysis (from a mean of 1170 admissions per year in the previous four years to 225 admissions between April 2020 through March 2021) [23]. A similar finding was observed for acute bronchiolitis among infants in Brazil—a significant reduction of more than 70% of hospitalizations was observed after the introduction of social distancing due to the COVID-19 pandemic [24]. Regarding emergency department (ED) utilization, a quasi-experimental interrupted time series analysis based on a French prospective surveillance system of six pediatric emergency departments demonstrated that significant decreases in ED visits (−79.7%, 95% CI, −84.3 to −73.8%; *p* < 0.0001), and hospital admissions (−71.3%, 95% CI, −78.8 to −61.1%; *p* < 0.0001) were observed for children with CAP [25]. A retrospective analysis of 29 acute care hospitals, covering 98% of ED beds in Israel found the same: that there was a decrease of 33% of ED visits for pneumonia and this reduction was consistently observed across all age groups [26]. All the above findings confirmed the decline in hospitalization utilization due to pneumonia and the major cause could be due to the indirect effect of NPI for the containment of COVID-19. However, the introduction of additional virtual services, and avoidance of exposure to the ED or hospital environment, could also result in a reduction in ED visits and hospitalization. In addition to respiratory tract infection, many other infections could be reduced in this pandemic. In Taiwan, where COVID-19 was well-controlled by aggressive infection prevention measures, 14 airborne/droplet, 11 fecal–oral, 7 vector-borne, and 4 direct contact transmitted notifiable infectious diseases had significant reductions of 2700 (−28.1%), 156 (−23.0%), 557 (−54.8%), and 73 (−45.9%) cases, respectively [27]. The similar findings were observed in Japan [28], China [29], Germany [30] and Australia [31]. Overall, these findings suggest that these NPI measurements such as masking, hand hygiene and social distancing during peaks of respiratory infections could be implemented in the future to help reduce respiratory morbidity and mortality.

## 3. Viral Respiratory Tract Infection

Since the outbreak of COVID-19, the distribution of causative pathogens in patients with respiratory tract infection has drastically changed. The virological distribution of respiratory virus underwent a huge shift due to SARS-CoV-2 becoming the most predominant virus causing LRTIs. In Germany, a study based on the prospective, multinational, multicenter cohort using molecular methods for the detection of the most common viral pathogens found that the most common pathogen was SARS-CoV-2 (96/435, 22%), followed by rhinovirus (24/435, 5.5%) and influenza virus (9/435, 2%) in 2020 [32]. This microbiological distribution is significantly different from those in 2018 and 2019, in which rhinovirus and influenza virus were the two most common pathogens [32]. Among pediatric patients with CAP in Shanghai, China, the detection rate of the eight common respiratory viruses including respiratory syncytial virus, influenza virus A and B, parainfluenza virus 1–3, adenovirus and human metapneumovirus from 2011 to 2019 ranged from 16.9% to 26.9%, but dramatically declined to 10.5% in 2020 [33]. The declining trend of respiratory virus, including influenza, parainfluenza virus, adenovirus, common cold coronavirus, human metapneumovirus, rhinovirus, and respiratory syncytial virus infection was consistently observed in another study in Texas, USA [34]. According to microbiological analysis among pediatric infections in Poland, there were 100% and 99% drops in influenza virus and respiratory syncytial virus, respectively, during the pandemic season until April 2021 in comparison to pre-pandemic years [23]. Finally, the significant decreases in seasonal influenza activity during COVID-19 have been observed in many countries, including the United States [35], Japan [6], England [36], Australia [35], Canada [37], South Africa [35], Singapore [38], Taiwan [39], South Korea [40], and Chile [35]. Overall, the viral distribution of respiratory tract infection has been largely shifted to the predominance of SARS-CoV-2, and the introduction of NPIs for COVID-19 could additionally help control the transmission of other SARS-CoV-2 respiratory viruses. Although previous studies have speculated the potential for both positive and negative interaction via an interferon-mediated mechanism between respiratory viruses [41,42], it remains unclear whether there is also the potential impact of viral–viral interactions between SARS-CoV-2 and other respiratory viruses on these declines.

## 4. Bacterial Pneumonia

Many studies demonstrated the change in incidence of bacterial pneumonia during the COVID-19 pandemic [19,43,44,45,46,47,48,49]. Although *S. pneumoniae* is the most common pathogen of CAP, the incidence of invasive pneumococcal disease (IPD) was significantly decreased in many countries during this pandemic [19,43,44,45,46,50,51,52]. In England, a prospective national cohort study revealed that the incidence of IPD in 2019/2020 was significantly lower than those in 2018/2019 (7.6/100,000 vs. 10.9/100,000; incidence rate ratio, 0.70; 95% CI, 0.18–2.67) [45]. In Hong Kong, the incidence of pneumococcal pneumonia and IPD in adult patients both significantly decreased by 82% and 89% (incidence rate ratios, 0.28 [95% CI, 0.23–0.33] and 0.11 [95% CI, 0.07–0.18]), respectively [19]. A similar trend was observed for elderly patients aged ≥65 years, in which there were reductions of 73% and 93% for pneumococcal pneumonia and IPD, respectively [19]. For children, a prospective cohort study in Israel showed that community-acquired alveolar pneumonia and bacteremic pneumococcal pneumonia were largely reduced by 93% and 81% (incidence rate ratios, 0.07 and 0.19, respectively) [43]. In the meanwhile, non-alveolar lower respiratory infections and non-pneumonia IPD were reduced, but the magnitude of reduction was only 54% and 58% (incidence rate ratios, 0.46 and 0.42, respectively) [43]. In South Korea, the incidence rate of IPD in children in 2020 decreased by 57%, compared with 2018 to 2019 (26.6 per 100,000 inpatients vs. 11.5 per 100,000 inpatients, *p* = 0.014) [44]. Moreover, a prospective analysis of surveillance data, laboratories in 26 countries and territories across 6 continents showed that all countries and territories had experienced a significant and sustained reduction in IPD in early 2020 (1 January to 31 May 2020) following the introduction of COVID-19 containment measures in each country [46]. In addition to *S. pneumoniae*, several studies also reported a decline in *H. influenzae* and *Mycoplasma pneumoniae* infection during COVID-19 pandemic [46,47,48]. However, several studies found that the increased presence of *Legionella* in the environment could be due to extreme stagnation in building water systems that resulted from prolonged building closures [53,54,55]. It may further increase the exposure risks to *Legionella* from building water systems during re-opening of previously closed buildings. Clinically, one study based on the Taiwan National Notifiable Disease Surveillance System found that compared with the pre-pandemic period, the rate of Legionnaires’ disease during the COVID-19 pandemic was increasing [49]. In summary, these findings urge us to establish an updated epidemiology of community-acquired bacterial pneumonia during the COVID-19 pandemic.

## 5. Tuberculosis (TB)

In addition to virus and bacteria, the outbreak of COVID-19 made a significant impact on the prevention, control and management of TB, particularly in developing countries [56]. The Global Tuberculosis Network collected data from 33 centers in 16 countries on 5 continents and showed that TB-related hospital discharges, newly diagnosed cases of active TB, total active TB outpatient visits, and new latent TB infection (LTBI) and LTBI outpatient visits were lower during the first 4 months of the pandemic in 2020 than for the corresponding period in 2019 [57]. Their findings [57] and many other studies [58,59,60,61,62,63,64,65] consistently indicated that COVID-19 has negatively impacted TB diagnosis, care, and prevention services globally. In contrast, the implementation of NPIs against SARS-CoV-2 outbreaks could potentially prevent the transmission of *Mycobacterium tuberculosis* (MTB). Like other respiratory pathogens, several studies reported that a reduction in respiratory contacts due to the widespread use of masks and social distancing may help reduce spread of MTB [27,66,67,68]. In summary, although the numbers of TB patients have declined globally during COVID-19 pandemic, the causes could be multifactorial, including the disruption of TB services and the prevention effects of NPIs. Further study is warranted to determine the key causes for TB declines. In the meantime, we should keep working on the restoration of TB diagnosis and care services for minimizing potentially negative effects on TB-related deaths.

## 6. Hospital-Acquired Pneumonia

Despite the fact that many NPIs were introduced into the healthcare system for the prevent and control of SARS-CoV-2, it was possible that the conventional infection prevention resources were shifted to focus on the COVID-19 response, particularly within the overwhelmed hospitals by the surging COVID-19 hospitalization [69]. The relaxed traditional infection control and prevention measures may change the epidemiology of healthcare-associated infections (HAIs), including HAP. One retrospective cohort study of mechanically ventilated adults at four academic and community hospitals in Massachusetts showed that ventilator-associated events (VAEs) per 100 episodes of mechanical ventilation were more common in 2020 than in prior years (11.2 vs. 6.7; *p* < 0.01). Although the rate of VAEs per 1000 ventilator days was also higher in 2020 than in previous years (14.2 vs. 12.7), the difference did not reach statistical significance (*p* = 0.08) [70]. Furthermore, another large US study based on the National Healthcare Safety Network demonstrated significant increases in the national standardized infection ratios for central-line-associated bloodstream infections (CLABSIs), catheter-associated urinary tract infections (CAUTIs), VAEs and MRSA bacteremia in 2020 [71]. In addition, the significant increases in the incidence of VAE were consistently observed across all four quarters of 2020 [71]. The condition could be worse for the patients with COVID-19. A study of 744 critically ill patients with COVID-19 reported that 759 episodes of HAIs developed in 359 patients, and VAP was the most common type of HAI (50%, *n* = 389) [72]. One survey showed that VAEs were more common in patients with COVID-19 than in patients without COVID-19 in 2020 (29.0 per 100 ventilator episodes vs. 7.1 per 100 ventilator episodes (*p* < 0.01) and 17.2 per 1000 ventilator days vs. 12.2 per 1000 ventilator days (*p* < 0.01)) [70]. Compared with inpatients without positive SARS-CoV-2, COVID-19 patients had significantly higher rates of infection-related ventilator-associated complications and probable VAP, with adjusted ORs of 4.7 (95% CI, 1.7–13.9) and 10.4 (95% CI, 2.1–52.1), respectively [73]. Among critical COVID-19 patients with VAP, Gram-negative bacteria and *Staphylococcus aureus* caused 64% and 28% of cases, respectively [72]. Overall, the updated meta-analysis of 34 studies involved 8901 cases, in which VAP was reported in 48.15% (95% CI, 42.3–54%) mechanically ventilated COVID-19 patients and the risk of VAP was higher in COVID-19 patients than other non-SARS-CoV-2 viral pneumonia (OR, 2.33; 95% CI, 1.75–3.11; *I*^2^ = 15%) [74]. All the above findings indicated the incidence of HAIs, including HAP, would increase during the COVID-19 pandemic and the risk of VAP could be higher in patients with critical COVID-19 than those with other non-COVID-19 pneumonia.

## 7. COVID-19 Co-Infection

In addition to non-COVID-19 pneumonia, clinicians should also be concerned about the possibility of COVID-19 co-infection. Many microorganisms, including bacteria, virus, fungi, and MTB, can cause co- or secondary infection with SARS-CoV-2 (Table 1) [75,76,77,78,79,80,81,82]. Based on the findings of a systemic review and meta-analysis including 64 studies with 61,547 patients, the estimated prevalence of co-infection was 16.98% (95% CI: 13.62–20.62%) [83]. The most common causative agents of co-infection among COVID-19 patients were bacteria (pooled prevalence: 20.97%; 95% CI: 15.95–26.46%), followed by virus (pooled prevalence: 12.58%; 95% CI: 7.31–18.96%) and fungus co-infections (pooled prevalence: 12.60%; 95% CI: 7.84–17.36%) [83]. Another meta-analysis including 118 articles demonstrated the similar findings—the pooled prevalence of co-infection was 19% (95% CI: 14–25%) [78]. However, they have different findings about the microbiologic distribution of COVID-19 co-infection—viral co-infection had the highest prevalence (the pooled prevalence: 10%; 95% CI: 6–14%), followed by bacteria (8%; 95% CI: 5–11%) and fungi (4%; 95% CI: 2–7%) [78].

Regarding specific pathogens, *K. pneumoniae* was the most common bacteria (9.9%), followed by *S. pneumoniae* (8.2%), *S. aureus* (7.7%), *H. influenza* (6.6%), and *M. pneumoniae* (3.8%) [78]. Among these bacteria, the role of MRSA lung infection in patients with COVID-19 cannot be neglected. There was significant heterogeneity in the retrieved literature on the epidemiology of MRSA pulmonary infection in patients with COVID-19. The relative prevalence ranged from 2% to 29% when considering all other bacteria as the denominator and the prevalence could increase to 11% to 65% when considering only *S. aureus* [84].

Among respiratory virus co-infection among patients with COVID-19, influenza A was the most common virus (22.3%), followed by influenza B (3.8%), respiratory syncytial virus (3.8%), rhinovirus (3.6%) and non-SARS-CoV-2 coronavirus (2.0%) [78]. One meta-analysis included 12 studies involving 9498 patients to investigate the impact of SARS-CoV-2 and influenza co-infection on disease outcomes and found that co-infection with influenza was not significantly associated with mortality (OR, 0.85; 95% CI: 0.51–1.43; *p* = 0.55, *I*^2^ = 76%) [85]. However, this result should be interpreted with caution due to its high heterogeneity, and a subgroup analysis with low heterogeneity found that mortality could significantly increase in studies outside of China (OR, 1.56; 95% CI: 1.12–2.19; *I*^2^ = 1%) [85].

Many fungi, including *Aspergillus*, *Candida* species, *Cryptococcus neoformans*, and fungi of the *Mucorales* order can cause co-infection with COVID-19 [82]. However, clinicians should be seriously concerned about two clinical entities—COVID-19 associated pulmonary aspergillosis (CAPA) and COVID-19 associated mucormycosis (CAM), which are aggressive fungal diseases with high morbidity and mortality [75,77]. One review reported that the incidence of CAPA ranged from 19.6% to 33.3% and the overall mortality was high, which could be up to 64.7% (*n* = 22) in the pooled analysis of 34 reported cases [77]. The use of corticosteroid and interleukin-6 blockade could be the significant risk factor of patients with CAPA [77]. The pooled prevalence of CAM was 7 cases per 1000 patients, which was 50 times higher than the highest recorded background of mucormycosis (0.14 cases per 1000 patients) [86]. Poor control of diabetes mellitus, such as diabetes ketoacidosis, is the most common risk factor of patients with CAM, and the mortality of patients with CAM was high with a pooled prevalence rate of 29.6% (95% CI, 17.2–45.9%) [86]. However, the diagnosis of both CAPA and CAM is not easy to make. It requires high index of suspicion and regular screening for risk factors and clinical features of them. Moreover, their treatment is complicated, which consists of controlling the underlying diseases (diabetes mellitus), withdrawal of immunomodulators, early antifungal therapy and extensive surgical debridement if needed [75,77].

However, the prevalence of co-infection among COVID-19 patients can vary according to the disease severity of SARS-CoV-2 infection, and diagnostic methods [76,83,87,88,89,90]. First, the prevalence of co-infection would increase with the disease severity of COVID-19. Severe/critical COVID-19 at presentation could be significantly and independently associated with the rate of co-infection association (adjusted OR, 4.42; 95% CI, 1.63–11.9) [91]. Second, the different diagnostic modality would have different detection rate of co-infections. One study compared the BioFire^®^ FilmArray^®^ Pneumonia Panel and conventional culture-based method to detect bacterial co-infection and found that the panel incidence of detections was 33% (95% CI, 0.25–0.41%, *I*^2^ = 32%), which was higher than the culture method of 18% (95% CI, 0.02–0.45%; *I*^2^ = 93%) [88]. Moreover, the molecular test could not only increase the rates of microbial detection but also significantly reduced the turn-around-time. Most importantly, there is robust evidence indicating that patients with co-infection would have worse outcomes, including longer length of hospital stays and higher risk of death, than those without co-infection [78,79,86,92,93]. Overall, this suggests that clinicians should keep alert to the possibility of co-infection among patients with COVID-19 and, further, make early diagnoses and apply appropriate antimicrobial treatment for this clinical entity.

## 8. Antibiotic Utilization and Resistance

Overuse of antibiotic for patients with SARS-CoV-2 infections during the first wave of COVID-19 due to the relaxed antimicrobial stewardship policies is common. One systemic review focusing on COVID-19 patients admitted to the ICU found that 71% (1929/2715) of them received antibiotics during their ICU stay [94]. Another meta-analysis involving 24 studies with 3338 patients with COVID-19 showed that 71.9% (95% CI 56.1–87.7%) of them received antibiotics but the overall proportion of COVID-19 patients with bacterial infection was 6.9% (95% CI 4.3–9.5%) [87]. Similar findings were reported in another meta-analysis including 154 studies—the prevalence of antibiotic prescribing was 74.6% (95% CI, 68.3–80.0%), which increased with patient age (OR, 1.45 per 10 year increase, 95% CI 1.18–1.77%) and the proportion of patients requiring mechanical ventilation (OR, 1.33 per 10% increase, 95% CI 1.15–1.54) [95]. In contrast, the estimated bacterial co-infection based on an analysis of 31 studies was only 8.6% (95% CI 4.7–15.2%) in this meta-analysis [95]. All these findings indicated that the prescribing is significantly higher than the estimated prevalence of bacterial co-infection and unnecessary antibiotic use could be high in patients with COVID-19.

In addition to the increasing use or overuse of antibiotics, we should seriously be concerned about another urgent threat to public health—the further development of antimicrobial resistance following inappropriate antibiotic use during the COVID-19 pandemic [96]. The association between the increasing use of antibiotics and antibiotic resistance has been demonstrated in several institutions [97,98]. A retrospective analysis in an intensive care unit in India showed that up to a 40% increase in antimicrobial resistance was observed amongst these isolated bacteria obtained during the COVID-19 period compared to pre-COVID-19 times [98]. A retrospective observational study in Brazil showed that the overall infection density of multidrug-resistant (MDR) organism infections significantly increased by 23% (*p* < 0.005) during COVID-19 [99] and the significant increases were observed for carbapenem-resistant *Acinetobacter baumannii* (CRAB) and MRSA in both ICU and non-ICU settings [99]. Furthermore, one study in an academic hospital reported that the acquisition of MDR Gram-negative bacteria would increase 3% for every increase in positive COVID-19 tests per week [100]. In the meantime, a significant spread of resistant pathogens, such as CRAB, carbapenem-resistant *P. aeruginosa*, carbapenem-resistant Enterobacterales, extended-spectrum β-lactamase-producing *K. pneumoniae*, MDR *E. coli* and vancomycin-resistant Enterococcus could develop among critically ill COVID-19 patients in the ICU [101,102,103,104,105,106,107]. In addition to antibiotic-resistant bacteria, MDR *Candida auris* should be monitored closely during this pandemic [108]. The cause of the increasing antimicrobial resistance could be multifactorial, including high antimicrobial exposure, environmental contamination, and disruption of infection control practice [105,109]. All these indicate that antibiotic stewardship programs remain essential to prevent unnecessary and inappropriate antibiotic use in hospitalized patients with COVID-19 and infection control interventions remain crucial to prevent the spread of MDR organisms.

## 9. Conclusions

**Figure 1:**
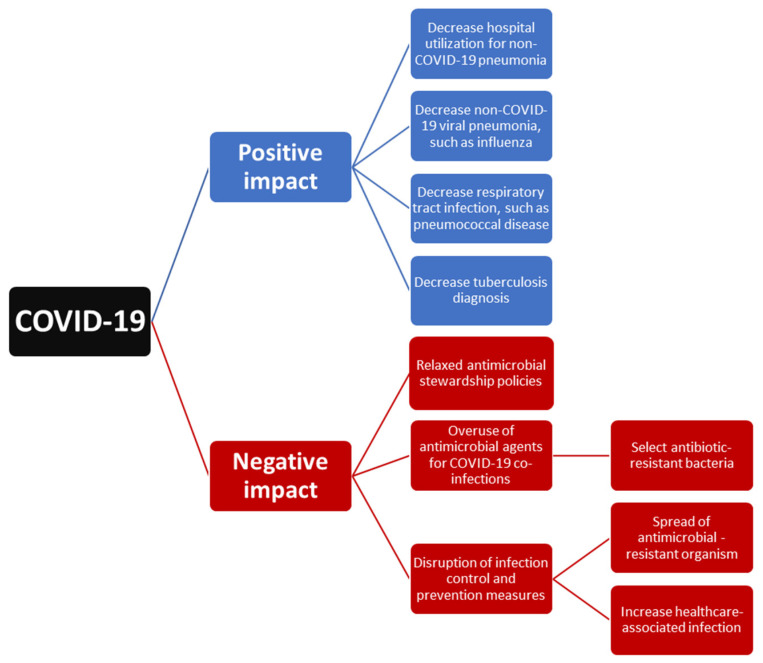
The impact of COVID-19 on the epidemiology of respiratory tract infection.

The outbreak of COVID-19 could exhibit both a positive impact due to the implementation of NPIs and a negative effect due to the disruption of healthcare systems at all levels (Figure 1). First, the implementation of NPIs for the containment of this outbreak also provides some additional benefit for global health. NPIs including universal masking, hand hygiene, and social distancing not only resulted in the decline in reported SARS-CoV-2 cases but also contributed to the decline in non-COVID-19 respiratory tract infection-related hospital utilization. Moreover, it led to the decreased incidence of previous commonly encountered respiratory pathogens, such as influenza and *S. pneumoniae.* However, the decline in TB could be attributed to the positive effect of NPIs and the negative impact of reduced TB service. Overall, our findings suggest that NPIs could be effective in preventing respiratory tract infection and changing the microbiologic distribution of respiratory pathogens. However, in this era of COVID-19, which is still evolving, we should keep conducting continuous epidemiological surveillance to monitor the trend of respiratory tract infection and further provide immediate information for making strategies to combat these infections.

Second, how to manage severe COVID-19 and its associated co-infection in acute and intensive care settings remains a great challenge. Although antimicrobial agents are an important treatment for patients with COVID-19 co-infection, many studies demonstrated that the prescribing of antibiotics was significantly higher than the estimated prevalence of bacterial co-infection and indicated the overuse of antibiotic or unnecessary antibiotic use during the COVID-19 pandemic. Moreover, inappropriate antimicrobial exposure may drive the selection of drug-resistant microorganisms and the disruption of infection control may result in the spread of drug-resistant pathogens. The threat of antimicrobial resistance will continue to pose a substantial impact on the healthcare systems. To mitigate the possible long-term impact of COVID-19 on antimicrobial resistance, it is necessary to integrate antimicrobial stewardship activities in the pandemic response to COVID-19 and develop novel approaches to stewardship according to COVID-19 settings and local epidemiology. In the meantime, close adherence to infection prevention and control measures is urgently needed.

## 10. Future Directions

Although COVID-19 drastically changed the whole world, the process of this fight against SARS-CoV-2 also taught us that we can do far more to prevent respiratory tract infection and its associated hospital utilization. At the same time, we should continue epidemiological surveillance, antimicrobial stewardship programs and infection control prevention interventions during this pandemic.

## Figures and Tables

**Table 1:** Microorganism can cause COVID-19 co-infection.

Microorganism	Pathogens
Bacteria	*Streptococcus pneumoniae*, *Staphylococcus aureus*, *Klebsiella pneumoniae*, *Mycoplasma pneumoniae*, *Chlamydophila pneumoniae*, *Legionella pneumophila*, *Escherichia coli*, *Stenotrophomonas maltophilia*, *Bordetella*, *Moraxella catarrhalis*, *Pseudomonas* spp., *Acinetobacter* spp. and *Enterococcus* spp.
Virus	non-SARS-CoV-2 coronavirus, influenza, respiratory syncytial virus, parainfluenza, rhinovirus, adenovirus and human immunodeficiency virus
Fungus	*Candida* spp., *Aspergillus* spp., *Rhizopus oryzae*, *Rhizopus microsporus*, *Rhizopus azygosporus*, *Lichtheimia mucor*, *Lichtheimia ramose*, and *Cryptococcus neoformans*
Mycobacterium	*Mycobacterium tuberculosis*

## References

[1] Lai C.C., Shih T.P., Ko W.C., Tang H.J., Hsueh P.R. (2020). Severe acute respiratory syndrome coronavirus 2 (SARS-CoV-2) and coronavirus disease-2019 (COVID-19): The epidemic and the challenges. Int. J. Antimicrob. Agents.

[2] World Health Organization. https://covid19.who.int/.

[3] Lai C.C., Chao C.M., Hsueh P.R. (2021). Clinical efficacy of antiviral agents against coronavirus disease 2019: A systematic review of randomized controlled trials. J. Microbiol. Immunol. Infect..

[4] Sun J., Shi Z., Xu H. (2020). Non-pharmaceutical interventions used for COVID-19 had a major impact on reducing influenza in China in 2020. J. Travel Med..

[5] Lai C.C., Yen M.Y., Lee P.I., Hsueh P.R. (2021). How to Keep COVID-19 at Bay: A Taiwanese Perspective. J. Epidemiol. Glob. Health.

[6] Sakamoto H., Ishikane M., Ueda P. (2020). Seasonal Influenza Activity During the SARS-CoV-2 Outbreak in Japan. JAMA.

[7] Chiu S.S., Cowling B.J., Peiris J.S.M., Chan E.L.Y., Wong W.H.S., Lee K.P. (2022). Effects of Nonpharmaceutical COVID-19 Interventions on Pediatric Hospitalizations for Other Respiratory Virus Infections, Hong Kong. Emerg. Infect. Dis..

[8] Lee H.H., Lin S.H. (2020). Effects of COVID-19 Prevention Measures on Other Common Infections, Taiwan. Emerg. Infect. Dis..

[9] Trenholme A., Webb R., Lawrence S., Arrol S., Taylor S., Ameratunga S., Byrnes C.A. (2021). COVID-19 and Infant Hospitalizations for Seasonal Respiratory Virus Infections, New Zealand, 2020. Emerg. Infect. Dis..

[10] Wong N.S., Leung C.C., Lee S.S. (2020). Abrupt Subsidence of Seasonal Influenza after COVID-19 Outbreak, Hong Kong, China. Emerg. Infect. Dis..

[11] Yum S., Hong K., Sohn S., Kim J., Chun B.C. (2021). Trends in Viral Respiratory Infections During COVID-19 Pandemic, South Korea. Emerg. Infect. Dis..

[12] Torres A., Cilloniz C., Niederman M.S., Menéndez R., Chalmers J.D., Wunderink R.G., van der Poll T. (2021). Pneumonia. Nat. Rev. Dis. Primers.

[13] GBD 2016 Lower Respiratory Infections Collaborators (2018). Estimates of the global, regional, and national morbidity, mortality, and aetiologies of lower respiratory infections in 195 countries, 1990-2016: A systematic analysis for the Global Burden of Disease Study 2016. Lancet Infect. Dis..

[14] GBD 2019 Diseases and Injuries Collaborators (2020). Global burden of 369 diseases and injuries in 204 countries and territories, 1990-2019: A systematic analysis for the Global Burden of Disease Study 2019. Lancet.

[15] Aliberti S., Dela Cruz C.S., Amati F., Sotgiu G., Restrepo M.I. (2021). Community-acquired pneumonia. Lancet.

[16] Cillóniz C., Ewig S., Polverino E., Marcos M.A., Esquinas C., Gabarrús A., Mensa J., Torres A. (2011). Microbial aetiology of community-acquired pneumonia and its relation to severity. Thorax.

[17] Torres A., Niederman M.S., Chastre J., Ewig S., Fernandez-Vandellos P., Hanberger H., Kollef M., Li Bassi G., Luna C.M., Martin-Loeches I. (2017). International ERS/ESICM/ESCMID/ALAT guidelines for the management of hospital-acquired pneumonia and ventilator-associated pneumonia: Guidelines for the management of hospital-acquired pneumonia (HAP)/ventilator-associated pneumonia (VAP) of the European Respiratory Society (ERS), European Society of Intensive Care Medicine (ESICM), European Society of Clinical Microbiology and Infectious Diseases (ESCMID) and Asociación Latinoamericana del Tórax (ALAT). Eur Respir J..

[18] Nagano H., Takada D., Shin J.H., Morishita T., Kunisawa S., Imanaka Y. (2021). Hospitalization of mild cases of community-acquired pneumonia decreased more than severe cases during the COVID-19 pandemic. Int. J. Infect. Dis..

[19] Chan K.F., Ma T.F., Ip M.S., Ho P.L. (2021). Invasive pneumococcal disease, pneumococcal pneumonia and all-cause pneumonia in Hong Kong during the COVID-19 pandemic compared with the preceding 5 years: A retrospective observational study. BMJ Open.

[20] Chiu N.C., Chi H., Tai Y.L., Peng C.C., Tseng C.Y., Chen C.C., Tan B.F., Lin C.Y. (2020). Impact of Wearing Masks, Hand Hygiene, and Social Distancing on Influenza, Enterovirus, and All-Cause Pneumonia During the Coronavirus Pandemic: Retrospective National Epidemiological Surveillance Study. J. Med. Internet Res..

[21] Lastrucci V., Bonaccorsi G., Forni S., D’Arienzo S., Bachini L., Paoli S., Lorini C., Gemmi F. (2021). The indirect impact of COVID-19 large-scale containment measures on the incidence of community-acquired pneumonia in older people: A region-wide population-based study in Tuscany, Italy. Int. J. Infect. Dis..

[22] Friedrich F., e Garcia L.D.C., Petry L.M., Pieta M.P., Carvalho G.E., Zocche G., Ongaratto R., Lumertz M.S., Brum M., Stein R.T. (2021). Impact of nonpharmacological COVID-19 interventions in hospitalizations for childhood pneumonia in Brazil. Pediatr. Pulmonol..

[23] Grochowska M., Ambrożej D., Wachnik A., Demkow U., Podsiadły E., Feleszko W. (2022). The Impact of the COVID-19 Pandemic Lockdown on Pediatric Infections-A Single-Center Retrospective Study. Microorganisms.

[24] Friedrich F., Ongaratto R., Scotta M.C., Veras T.N., Stein R.T., Lumertz M.S., Jones M.H., Comaru T., Pinto L.A. (2021). Early Impact of Social Distancing in Response to Coronavirus Disease 2019 on Hospitalizations for Acute Bronchiolitis in Infants in Brazil. Clin. Infect. Dis..

[25] Rybak A., Yang D.D., Schrimpf C., Guedj R., Levy C., Cohen R., Gajdos V., Tort J., Skurnik D., Ouldali N. (2021). Fall of Community-Acquired Pneumonia in Children following COVID-19 Non-Pharmaceutical Interventions: A Time Series Analysis. Pathogens.

[26] Haklai Z., Applbaum Y., Myers V., Saban M., Gordon E.S., Luxenburg O., Wilf-Miron R. (2022). The effect of the COVID-19 pandemic on non-COVID respiratory ED visits in Israel. Am. J. Emerg. Med..

[27] Lai C.C., Chen S.Y., Yen M.Y., Lee P.I., Ko W.C., Hsueh P.R. (2021). The impact of the coronavirus disease 2019 epidemic on notifiable infectious diseases in Taiwan: A database analysis. Travel Med. Infect. Dis..

[28] Hibiya K., Iwata H., Kinjo T., Shinzato A., Tateyama M., Ueda S., Fujita J. (2022). Incidence of common infectious diseases in Japan during the COVID-19 pandemic. PLoS ONE.

[29] Chen B., Wang M., Huang X., Xie M., Pan L., Liu H., Liu Z., Zhou P. (2021). Changes in Incidence of Notifiable Infectious Diseases in China Under the Prevention and Control Measures of COVID-19. Front. Public Health.

[30] Ullrich A., Schranz M., Rexroth U., Hamouda O., Schaade L., Diercke M., Boender T.S. (2021). Impact of the COVID-19 pandemic and associated non-pharmaceutical interventions on other notifiable infectious diseases in Germany: An analysis of national surveillance data during week 1-2016–week 32-2020. Lancet Reg. Health Eur..

[31] Adegbija O., Walker J., Smoll N., Khan A., Graham J., Khandaker G. (2021). Notifiable diseases after implementation of COVID-19 public health prevention measures in Central Queensland, Australia. Commun. Dis. Intell..

[32] Dähne T., Bauer W., Essig A., Schaaf B., Spinner C.D., Pletz M.W., Rohde G., Rupp J., Witzenrath M., Panning M. (2021). The impact of the SARS-CoV-2 pandemic on the prevalence of respiratory tract pathogens in patients with community-acquired pneumonia in Germany. Emerg. Microbes Infect..

[33] Li F., Zhang Y., Shi P., Cao L., Su L., Zhang Y., Peng K., Lu R., Tan W., Shen J. (2021). Epidemiology of Viruses Causing Pediatric Community Acquired Pneumonia in Shanghai During 2010-2020: What Happened Before and After the COVID-19 Outbreak?. Infect. Dis Ther..

[34] Mutnal M.B., Arroliga A.C., Walker K., Mohammad A., Brigmon M.M., Beaver R.M., Midturi J.K., Rao A. (2020). Early trends for SARS-CoV-2 infection in central and north Texas and impact on other circulating respiratory viruses. J. Med. Virol..

[35] Olsen S.J., Azziz-Baumgartner E., Budd A.P., Brammer L., Sullivan S., Pineda R.F., Cohen C., Fry A.M. (2020). Decreased Influenza Activity During the COVID-19 Pandemic—United States, Australia, Chile, and South Africa, 2020. MMWR Morb. Mortal Wkly. Rep..

[36] Kadambari S., Goldacre R., Morris E., Goldacre M.J., Pollard A.J. (2022). Indirect effects of the covid-19 pandemic on childhood infection in England: Population based observational study. BMJ.

[37] Lee L., Butt K., Buckrell S., Nwosu A., Sevenhuysen C., Bancej C. (2021). National influenza mid-season report, 2020–2021. Can. Commun. Dis. Rep..

[38] Soo R.J.J., Chiew C.J., Ma S., Pung R., Lee V. (2020). Decreased Influenza Incidence under COVID-19 Control Measures, Singapore. Emerg. Infect. Dis..

[39] Kuo S.C., Shih S.M., Chien L.H., Hsiung C.A. (2020). Collateral Benefit of COVID-19 Control Measures on Influenza Activity, Taiwan. Emerg. Infect. Dis..

[40] Lee H., Lee H., Song K.H., Kim E.S., Park J.S., Jung J., Ahn S., Jeong E.K., Park H., Kim H.B. (2021). Impact of Public Health Interventions on Seasonal Influenza Activity During the COVID-19 Outbreak in Korea. Clin. Infect. Dis..

[41] Nickbakhsh S., Mair C., Matthews L., Reeve R., Johnson P.C.D., Thorburn F., von Wissmann B., Reynolds A., McMenamin J., Gunson R.N. (2019). Virus-virus interactions impact the population dynamics of influenza and the common cold. Proc. Natl. Acad. Sci. USA.

[42] Wu A., Mihaylova V.T., Landry M.L., Foxman E.F. (2020). Interference between rhinovirus and influenza A virus: A clinical data analysis and experimental infection study. Lancet Microbe.

[43] Danino D., Ben-Shimol S., Van Der Beek B.A., Givon-Lavi N., Avni Y.S., Greenberg D., Weinberger D.M., Dagan R. (2021). Decline in Pneumococcal Disease in Young Children during the COVID-19 Pandemic in Israel Associated with Suppression of seasonal Respiratory Viruses, despite Persistent Pneumococcal Carriage: A Prospective Cohort Study. Clin. Infect. Dis..

[44] Kim Y.K., Choi Y.Y., Lee H., Song E.S., Ahn J.G., Park S.E., Lee T., Cho H.K., Lee J., Kim Y.J. (2022). Differential Impact of Nonpharmaceutical Interventions on the Epidemiology of Invasive Bacterial Infections in Children During the Coronavirus Disease 2019 Pandemic. Pediatr. Infect. Dis. J..

[45] Amin-Chowdhury Z., Aiano F., Mensah A., Sheppard C.L., Litt D., Fry N.K., Andrews N., Ramsay M.E., Ladhani S.N. (2021). Impact of the Coronavirus Disease 2019 (COVID-19) Pandemic on Invasive Pneumococcal Disease and Risk of Pneumococcal Coinfection with Severe Acute Respiratory Syndrome Coronavirus 2 (SARS-CoV-2): Prospective National Cohort Study, England. Clin. Infect. Dis.

[46] Brueggemann A.B., Jansen van Rensburg M.J., Shaw D., McCarthy N.D., Jolley K.A., Maiden M.C.J., van der Linden M.P.G., Amin-Chowdhury Z., Bennett D.E., Borrow R. (2021). Changes in the incidence of invasive disease due to *Streptococcus pneumoniae*, Haemophilus influenzae, and Neisseria meningitidis during the COVID-19 pandemic in 26 countries and territories in the Invasive Respiratory Infection Surveillance Initiative: A prospective analysis of surveillance data. Lancet Digit. Health.

[47] Zhang Y., Huang Y., Ai T., Luo J., Liu H. (2021). Effect of COVID-19 on childhood Mycoplasma pneumoniae infection in Chengdu, China. BMC Pediatr..

[48] Fujita J. (2021). Mycoplasma pneumoniae pneumonia and respiratory syncytial virus infection in Japan during the severe acute respiratory syndrome coronavirus 2 pandemic. Respir. Investig..

[49] Chao C.M., Lai C.C. (2022). Increasing legionella in Taiwan during COVID-19 pandemic. Am. J. Infect. Control..

[50] Huh K., Jung J., Hong J., Kim M., Ahn J.G., Kim J.H., Kang J.M. (2021). Impact of Nonpharmaceutical Interventions on the Incidence of Respiratory Infections During the Coronavirus Disease 2019 (COVID-19) Outbreak in Korea: A Nationwide Surveillance Study. Clin. Infect. Dis..

[51] Juan H.C., Chao C.M., Lai C.C., Tang H.J. (2021). Decline in invasive pneumococcal disease during COVID-19 pandemic in Taiwan. J. Infect..

[52] Lim R.H., Chow A., Ho H.J. (2020). Decline in pneumococcal disease incidence in the time of COVID-19 in Singapore. J. Infect..

[53] Liang J., Swanson C.S., Wang L., He Q. (2021). Impact of building closures during the COVID-19 pandemic on Legionella infection risks. Am. J. Infect. Control..

[54] Palazzolo C., Maffongelli G., D’Abramo A., Lepore L., Mariano A., Vulcano A., Bartoli T.A., Bevilacqua N., Giancola M.L., Di Rosa E. (2020). Legionella pneumonia: Increased risk after COVID-19 lockdown? Italy, May to June 2020. Euro. Surveill..

[55] De Giglio O., Diella G., Lopuzzo M., Triggiano F., Calia C., Pousis C., Fasano F., Caggiano G., Calabrese G., Rafaschieri V. (2020). Impact of lockdown on the microbiological status of the hospital water network during COVID-19 pandemic. Environ. Res..

[56] Alene K.A., Wangdi K., Clements A.C.A. (2020). Impact of the COVID-19 Pandemic on Tuberculosis Control: An Overview. Trop Med. Infect. Dis..

[57] Migliori G.B., Thong P.M., Akkerman O., Alffenaar J.W., Álvarez-Navascués F., Assao-Neino M.M., Bernard P.V., Biala J.S., Blanc F.X., Bogorodskaya E.M. (2020). Worldwide Effects of Coronavirus Disease Pandemic on Tuberculosis Services, January-April 2020. Emerg. Infect. Dis..

[58] Nikolayevskyy V., Holicka Y., van Soolingen D., van der Werf M.J., Ködmön C., Surkova E., Hillemann D., Groenheit R., Cirillo D. (2021). Impact of the COVID-19 pandemic on tuberculosis laboratory services in Europe. Eur. Respir. J..

[59] Magro P., Formenti B., Marchese V., Gulletta M., Tomasoni L.R., Caligaris S., Castelli F., Matteelli A. (2020). Impact of the SARS-CoV-2 epidemic on tuberculosis treatment outcome in Northern Italy. Eur. Respir. J..

[60] Odume B., Falokun V., Chukwuogo O., Ogbudebe C., Useni S., Nwokoye N., Aniwada E., Olusola Faleye B., Okekearu I., Nongo D. (2020). Impact of COVID-19 on TB active case finding in Nigeria. Public Health Action.

[61] Jain V.K., Iyengar K.P., Samy D.A., Vaishya R. (2020). Tuberculosis in the era of COVID-19 in India. Diabetes Metab. Syndr..

[62] Caren G.J., Iskandar D., Pitaloka D.A.E., Abdulah R., Suwantika A.A. (2022). COVID-19 Pandemic Disruption on the Management of Tuberculosis Treatment in Indonesia. J. Multidiscip. Healthc..

[63] Min J., Ko Y., Kim H.W., Koo H.K., Oh J.Y., Jeong Y.J., Kang H.H., Park K.J., Hwang Y.I., Kim J.W. (2022). Increased Healthcare Delays in Tuberculosis Patients During the First Wave of COVID-19 Pandemic in Korea: A Nationwide Cross-Sectional Study. J. Korean Med. Sci..

[64] Geric C., Saroufim M., Landsman D., Richard J., Benedetti A., Batt J., Brode S.K., Ahmad Khan F. (2021). Impact of Covid-19 on Tuberculosis Prevention and Treatment in Canada: A multicentre analysis of 10,833 patients. J. Infect. Dis..

[65] Dara M., Kuchukhidze G., Yedilbayev A., Perehinets I., Schmidt T., Van Grinsven W.L., Boeree M.J. (2021). Early COVID-19 pandemic’s toll on tuberculosis services, WHO European Region, January to June 2020. Euro. Surveill..

[66] Ding W., Li Y., Bai Y., Li Y., Wang L., Wang Y. (2021). Estimating the Effects of the COVID-19 Outbreak on the Reductions in Tuberculosis Cases and the Epidemiological Trends in China: A Causal Impact Analysis. Infect. Drug Resist..

[67] Beyene N.W., Sitotaw A.L., Tegegn B., Bobosha K. (2021). The impact of COVID-19 on the tuberculosis control activities in Addis Ababa. Pan. Afr. Med. J..

[68] Fang J.L., Chao C.M., Tang H.J. (2020). The impact of COVID-19 on the diagnosis of TB in Taiwan. Int. J. Tuberc. Lung Dis..

[69] Stevens M.P., Doll M., Pryor R., Godbout E., Cooper K., Bearman G. (2020). Impact of COVID-19 on traditional healthcare-associated infection prevention efforts. Infect. Control. Hosp. Epidemiol..

[70] Weinberger J., Rhee C., Klompas M. (2022). Incidence, Characteristics, and Outcomes of Ventilator-associated Events during the COVID-19 Pandemic. Ann. Am. Thorac. Soc..

[71] Weiner-Lastinger L.M., Pattabiraman V., Konnor R.Y., Patel P.R., Wong E., Xu S.Y., Smith B., Edwards J.R., Dudeck M.A. (2022). The impact of coronavirus disease 2019 (COVID-19) on healthcare-associated infections in 2020: A summary of data reported to the National Healthcare Safety Network. Infect. Control. Hosp. Epidemiol..

[72] Grasselli G., Scaravilli V., Mangioni D., Scudeller L., Alagna L., Bartoletti M., Bellani G., Biagioni E., Bonfanti P., Bottino N. (2021). Hospital-Acquired Infections in Critically Ill Patients With COVID-19. Chest.

[73] DeVoe C., Segal M.R., Wang L., Stanley K., Madera S., Fan J., Schouest J., Graham-Ojo R., Nichols A., Prasad P.A. (2021). Increased rates of secondary bacterial infections, including Enterococcus bacteremia, in patients hospitalized with coronavirus disease 2019 (COVID-19). Infect. Control. Hosp. Epidemiol..

[74] Jain S., Khanna P., Sarkar S. (2021). Comparative evaluation of ventilator-associated pneumonia in critically ill COVID- 19 and patients infected with other corona viruses: A systematic review and meta-analysis. Monaldi. Arch. Chest Dis..

[75] Chao C.M., Lai C.C., Yu W.L. (2022). COVID-19 associated mucormycosis—An emerging threat. J. Microbiol. Immunol. Infect..

[76] Lai C.C., Wang C.Y., Hsueh P.R. (2020). Co-infections among patients with COVID-19: The need for combination therapy with non-anti-SARS-CoV-2 agents?. J. Microbiol. Immunol. Infect..

[77] Lai C.C., Yu W.L. (2021). COVID-19 associated with pulmonary aspergillosis: A literature review. J. Microbiol. Immunol. Infect..

[78] Musuuza J.S., Watson L., Parmasad V., Putman-Buehler N., Christensen L., Safdar N. (2021). Prevalence and outcomes of co-infection and superinfection with SARS-CoV-2 and other pathogens: A systematic review and meta-analysis. PLoS ONE.

[79] Song W.M., Zhao J.Y., Zhang Q.Y., Liu S.Q., Zhu X.H., An Q.Q., Xu T.T., Li S.J., Liu J.Y., Tao N.N. (2021). COVID-19 and Tuberculosis Coinfection: An Overview of Case Reports/Case Series and Meta-Analysis. Front. Med..

[80] Hedberg P., Johansson N., Ternhag A., Abdel-Halim L., Hedlund J., Nauclér P. (2022). Bacterial co-infections in community-acquired pneumonia caused by SARS-CoV-2, influenza virus and respiratory syncytial virus. BMC Infect. Dis..

[81] Kılıç L., Altın S., Gönenç Ortaköylü M., Kanmaz Z.D., Tutar T., Özkan G.Z. (2022). Co-infection of COVID-19 and Tuberculosis. Turk. Thorac. J..

[82] Amin A., Vartanian A., Poladian N., Voloshko A., Yegiazaryan A., Al-Kassir A.L., Venketaraman V. (2021). Root Causes of Fungal Coinfections in COVID-19 Infected Patients. Infect. Dis. Rep..

[83] Pakzad R., Malekifar P., Shateri Z., Zandi M., Akhavan Rezayat S., Soleymani M., Karimi M.R., Ahmadi S.E., Shahbahrami R., Pakzad I. (2022). Worldwide prevalence of microbial agents’ coinfection among COVID-19 patients: A comprehensive updated systematic review and meta-analysis. J. Clin. Lab. Anal..

[84] Bassetti M., Magnasco L., Vena A., Portunato F., Giacobbe D.R. (2022). Methicillin-resistant Staphylococcus aureus lung infection in coronavirus disease 2019: How common?. Curr. Opin. Infect. Dis..

[85] Guan Z., Chen C., Li Y., Yan D., Zhang X., Jiang D., Yang S., Li L. (2021). Impact of Coinfection With SARS-CoV-2 and Influenza on Disease Severity: A Systematic Review and Meta-Analysis. Front. Public Health.

[86] Hussain S., Riad A., Singh A., Klugarová J., Antony B., Banna H., Klugar M. (2021). Global Prevalence of COVID-19-Associated Mucormycosis (CAM): Living Systematic Review and Meta-Analysis. J. Fungi.

[87] Langford B.J., So M., Raybardhan S., Leung V., Westwood D., MacFadden D.R., Soucy J.R., Daneman N. (2020). Bacterial co-infection and secondary infection in patients with COVID-19: A living rapid review and meta-analysis. Clin. Microbiol. Infect..

[88] Timbrook T.T., Hueth K.D., Ginocchio C.C. (2021). Identification of bacterial co-detections in COVID-19 critically Ill patients by BioFire^®^ FilmArray^®^ pneumonia panel: A systematic review and meta-analysis. Diagn. Microbiol. Infect. Dis..

[89] Soltani S., Faramarzi S., Zandi M., Shahbahrami R., Jafarpour A., Akhavan Rezayat S., Pakzad I., Abdi F., Malekifar P., Pakzad R. (2021). Bacterial coinfection among coronavirus disease 2019 patient groups: An updated systematic review and meta-analysis. New Microbes. New Infect..

[90] Lansbury L., Lim B., Baskaran V., Lim W.S. (2020). Co-infections in people with COVID-19: A systematic review and meta-analysis. J. Infect..

[91] Nasir N., Rehman F., Omair S.F. (2021). Risk factors for bacterial infections in patients with moderate to severe COVID-19: A case-control study. J. Med. Virol..

[92] Danwang C., Noubiap J.J., Robert A., Yombi J.C. (2022). Outcomes of patients with HIV and COVID-19 co-infection: A systematic review and meta-analysis. AIDS Res. Ther..

[93] Sarkar S., Khanna P., Singh A.K. (2021). Impact of COVID-19 in patients with concurrent co-infections: A systematic review and meta-analyses. J. Med. Virol..

[94] Abu-Rub L.I., Abdelrahman H.A., Johar A.A., Alhussain H.A., Hadi H.A., Eltai N.O. (2021). Antibiotics Prescribing in Intensive Care Settings during the COVID-19 Era: A Systematic Review. Antibiotics.

[95] Langford B.J., So M., Raybardhan S., Leung V., Soucy J.R., Westwood D., Daneman N., MacFadden D.R. (2021). Antibiotic prescribing in patients with COVID-19: Rapid review and meta-analysis. Clin. Microbiol. Infect..

[96] Magnasco L., Mikulska M., Giacobbe D.R., Taramasso L., Vena A., Dentone C., Dettori S., Tutino S., Labate L., Di Pilato V. (2021). Spread of Carbapenem-Resistant Gram-Negatives and Candida auris during the COVID-19 Pandemic in Critically Ill Patients: One Step Back in Antimicrobial Stewardship?. Microorganisms.

[97] Lai C.C., Chen S.Y., Ko W.C., Hsueh P.R. (2021). Increased antimicrobial resistance during the COVID-19 pandemic. Int. J. Antimicrob. Agents.

[98] Saini V., Jain C., Singh N.P., Alsulimani A., Gupta C., Dar S.A., Haque S., Das S. (2021). Paradigm Shift in Antimicrobial Resistance Pattern of Bacterial Isolates during the COVID-19 Pandemic. Antibiotics.

[99] Polly M., de Almeida B.L., Lennon R.P., Cortês M.F., Costa S.F., Guimarães T. (2022). Impact of the COVID-19 pandemic on the incidence of multidrug-resistant bacterial infections in an acute care hospital in Brazil. Am. J. Infect. Control..

[100] Bork J.T., Leekha S., Claeys K., Seung H., Tripoli M., Amoroso A., Heil E.L. (2021). Change in hospital antibiotic use and acquisition of multidrug-resistant gram-negative organisms after the onset of coronavirus disease 2019. Infect. Control. Hosp. Epidemiol..

[101] Thoma R., Seneghini M., Seiffert S.N., Vuichard Gysin D., Scanferla G., Haller S., Flury D., Boggian K., Kleger G.R., Filipovic M. (2022). The challenge of preventing and containing outbreaks of multidrug-resistant organisms and Candida auris during the coronavirus disease 2019 pandemic: Report of a carbapenem-resistant Acinetobacter baumannii outbreak and a systematic review of the literature. Antimicrob. Resist. Infect. Control..

[102] Tiri B., Sensi E., Marsiliani V., Cantarini M., Priante G., Vernelli C., Martella L.A., Costantini M., Mariottini A., Andreani P. (2020). Antimicrobial Stewardship Program, COVID-19, and Infection Control: Spread of Carbapenem-Resistant Klebsiella Pneumoniae Colonization in ICU COVID-19 Patients. What Did Not Work?. J. Clin. Med..

[103] Emeraud C., Figueiredo S., Bonnin R.A., Khecharem M., Ouzani S., Leblanc P.E., Jousset A.B., Fortineau N., Duranteau J., Dortet L. (2021). Outbreak of CTX-M-15 Extended-Spectrum β-Lactamase-Producing Klebsiella pneumoniae ST394 in a French Intensive Care Unit Dedicated to COVID-19. Pathogens.

[104] Perez S., Innes G.K., Walters M.S., Mehr J., Arias J., Greeley R., Chew D. (2020). Increase in Hospital-Acquired Carbapenem-Resistant Acinetobacter baumannii Infection and Colonization in an Acute Care Hospital During a Surge in COVID-19 Admissions—New Jersey, February-July 2020. MMWR Morb. Mortal Wkly. Rep..

[105] Patel A., Emerick M., Cabunoc M.K., Williams M.H., Preas M.A., Schrank G., Rabinowitz R., Luethy P., Johnson J.K., Leekha S. (2021). Rapid Spread and Control of Multidrug-Resistant Gram-Negative Bacteria in COVID-19 Patient Care Units. Emerg. Infect. Dis..

[106] García-Meniño I., Forcelledo L., Rosete Y., García-Prieto E., Escudero D., Fernández J. (2021). Spread of OXA-48-producing Klebsiella pneumoniae among COVID-19-infected patients: The storm after the storm. J. Infect. Public Health.

[107] Kampmeier S., Tönnies H., Correa-Martinez C.L., Mellmann A., Schwierzeck V. (2020). A nosocomial cluster of vancomycin resistant enterococci among COVID-19 patients in an intensive care unit. Antimicrob. Resist. Infect. Control..

[108] Janniger E.J., Kapila R. (2021). Public health issues with Candida auris in COVID-19 patients. World Med. Health Policy.

[109] Segala F.V., Bavaro D.F., Di Gennaro F., Salvati F., Marotta C., Saracino A., Murri R., Fantoni M. (2021). Impact of SARS-CoV-2 Epidemic on Antimicrobial Resistance: A Literature Review. Viruses.

